# Endoscopic treatment outcomes for foreign body extraction from alimentary tract: a 15-year experience of 1,162 cases in Southern China

**DOI:** 10.3389/fmed.2026.1877577

**Published:** 2026-07-01

**Authors:** Weidong Feng, Jian Zhang, Yufeng Ren, Zhen Ding, Yujie Yuan

**Affiliations:** 1 Center of Gastrointestinal Surgery, The First Affiliated Hospital of Sun Yat-sen University, Guangzhou, China; 2 Endoscopy Center, The First Affiliated Hospital of Sun Yat-sen University, Guangzhou, China; 3 Center of Digestive Medicine, The Seventh Affiliated Hospital of Sun Yat-sen University, Shenzhen, China; 4 Department of General Surgery, Linzhi People's Hospital, Linzhi, China; 5 Department of Radiation Oncology, The First Affiliated Hospital, Sun Yat-sen University, Guangzhou, China; 6 Jieyang Cancer Center, Jieyang People's Hospital, Jieyang, Guangdong, China

**Keywords:** endoscopy, foreign bodies, gastrointestinal tract, outcomes, risk factors

## Abstract

**Background:**

Endoscopic removal of foreign bodies (FB) impacted in the alimentary tract represents a critical and technically challenging emergency scenario. This retrospective study aimed to evaluate the clinical outcomes of endoscopic intervention for FB removal and to identify independent risk factors associated with procedural failure.

**Methods:**

A single-center retrospective analysis was conducted on consecutive patients with a confirmed diagnosis of FB impaction in the upper or lower gastrointestinal (GI) tract between January 2010 and December 2024. Baseline demographics, clinical presentations, and endoscopic characteristics were reviewed. The success rate of endoscopic retrieval, along with procedure-related complication rates, were investigated. Independent predictors of endoscopic failure were identified using multivariable logistic regression analysis.

**Results:**

A total of 1,162 cases were analyzed (51.3% male; 55.6% aged ≥60 years). The vast majority of FBs were located in the upper GI tract (97.3%) and resulted from unwitnessed or accidental ingestions (89.7%). Endoscopic removal was successful in 1140 cases (98.1%), with 84.7% completed within 24 h of symptom onset. The incidence of moderate-to-severe complications was 0.6%, with no death related to FB impaction or endoscopic intervention. Independent risk factors for endoscopic failure included sharp pointed FB (*p* = 0.014), delayed intervention exceeding 24 h (*p* = 0.035) and multiple endoscopic attempts (*p* < 0.001).

**Conclusion:**

Endoscopic management of GI tract FB impaction is highly effective and carries a favorable safety profile. Early recognition of factors predictive of endoscopic failure may enhance the success rate of FB removal in emergency situations.

## Introduction

Foreign bodies (FB) impaction in the alimentary tract represents a frequent and critical clinical emergency. This condition predominantly occurs in children and older adults aged 65 years and above, often resulting from accidental or unconscious ingestion, with the esophagus serving as the most frequently involved anatomical site (1, 2). Additionally, the clinical burden of lower gastrointestinal (GI) tract involvement is rising; the annual incidence of colorectal FB increased from 1.2 to 1.9 per 100,000 individuals in the United States between 2012 and 2021, with 40.8% of these cases requiring hospitalization for further medical intervention (3).

FB impaction in the gut could be resolved most of time through emergent treatment including endoscopic and surgical retraction. Since its pioneering description by McKechnie et al. (4), endoscopic removal has established itself as the gold-standard modality for extracting FBs from the upper GI tract. Subsequent literature has consistently demonstrated that endoscopy achieves remarkably high success rates, ranging from 95.0 to 98.8%, for removing FB from the upper GI tract, paired with low complication rates spanning only 0 to 5% (5, 6).

This study aims to summarize the long-term outcomes following endoscopic treatment of FB impaction throughout the entire GI tract. Meanwhile, this study also aims to identify independent risk factors associated with endoscopic procedural failure, which may risk-stratify patients and guide clinicians in proactively selecting safer, less invasive alternatives to emergency surgery. To our knowledge, this is the largest single-center cohort report, and it includes lower GI cases that have been largely excluded from prior major studies.

## Materials and methods

### Patient selection

This was a single-center, retrospective study conducted between January 2010 and December 2024. Patients who were admitted or referred to our institution for suspected FB impaction were initially identified from our electronic endoscopic database. After that, all eligible cases were reviewed by two experienced endoscopists (YY and WF) by extracting specific information from electronic medical records. Missing data was managed with the listwise deletion method. No restrictions were applied regarding patient age, sex, BMI, or pre-existing comorbidities during initial screening. Following the exclusion of cases involving FBs within the respiratory tract, primary surgical intervention without endoscopic removal attempt, misdiagnosis of FB impaction, or a lack of core clinical data, a final cohort of 1,162 cases was enrolled in the analysis. The study followed the STROBE guideline and was approved by our institutional review board. Due to the retrospective design of the study, the requirement for patient informed consent for data review was waived.

### Study design

Prior to the endoscopic procedure, patients with a confirmed diagnosis of FB impaction in the upper or lower GI tract received topical or general anesthesia. Topical anesthesia was achieved using lidocaine hydrochloride gel. General anesthesia was typically induced via propofol administration by trained anesthesiologists. All endoscopic FB retrievals were performed by on-duty endoscopists and their supervising attending physicians, who managed any intraoperative emergencies. The choice between a flexible gastroscope or colonoscope was dictated by the anatomical location of the FB. A variety of accessory devices including FB retrieval forceps, biopsy forceps, retrieval baskets, nets and snares were selected based on the nature of the FB and the endoscopist’s preference or prior experience. Additionally, transparent caps or over-tubes were applied to minimize mucosal or transmural injury during the extraction process. Cardiopulmonary function of patients was monitored with pulse oximetry, with supplemental nasal oxygen therapy supplied on demand. All patients provided written informed consent prior to undergoing the endoscopic intervention.

In accordance with previously established criteria (1, 7), FBs were classified into three morphological groups (Appendix Figure 1): (1) Blunt objects: coins, buttons, toys, battery, magnetic bead, etc.; (2) Sharp-pointed objects: bones, needles, toothpicks, glass pieces, drug shell, etc.; and (3) Food boluses. The size and anatomical location of each impacted FB were recorded, alongside corresponding procedural outcomes and treatment-related complications.

For patients who did not respond to endoscopic treatment, rescue surgery via emergent thoracic or abdominal exploration was performed by surgeons at our institution. Patients presenting with severe symptoms caused by FB stuck in the small bowel underwent emergent laparotomy and were excluded from this study.

The following demographic, clinical and endoscopic parameters were obtained from the medical records and endoscopic datasets: (1) Demographic Data: age, sex, etiology of FB impaction, symptoms, comorbidities; (2) Clinical Data: FB type, FB location, FB size (maximum diameter estimated endoscopically or measured via CT scans), number and duration of FB impaction, 30-day mortality rate, and the rate of rescue operations due to endoscopic failure; and (3) Endoscopic Data: time of endoscopic intervention, anesthesia method, FB retrieval device, times of endoscopic attempts, endoscopic outcome (success or failure) and associated complications.

Endoscopic complications were defined as any adverse event directly related to the endoscopic procedure that required unscheduled or prolonged medical treatment, including hemorrhage, perforation (transmural tearing of the digestive tract), adverse reactions to sedation, or cardiopulmonary events. Conversely, complications secondary to the FB impaction itself were defined as perforation driven by FB migration, or localized inflammation/abscess formation due to prolong FB stuck *in situ*.

### Primary and secondary outcomes

The primary outcome of this study was the technical success rate of endoscopic FB retrieval from the GI tract. Secondary outcomes included endoscopic complications, the rate of rescue surgery due to unsuccessful endoscopic treatment, and short-term clinical outcomes which included length of hospital stay and 30-day mortality rate. Additionally, independent risk factors associated with endoscopic failure were identified via multivariate logistic regression analysis.

### Statistical analysis

Descriptive statistics were expressed as frequencies and percentages for categorical variables unless specified otherwise. To identify independent predictors of endoscopic failure, variables demonstrating a trend toward association (*p* < 0.20) in the initial univariate logistic regression analysis were entered into a multivariate logistic regression model using endoscopic failure as the dependent variable. All statistical analyses were performed using STATA 18.0 (Stata Corporation, College Station, TX, United States) and GraphPad Prism 10.0 (GraphPad Software Inc., San Diego, CA, United States), with a two-sided *p* value <0.05 indicating statistically significant.

## Results

### Baseline patient characteristics

**Figure 1:**
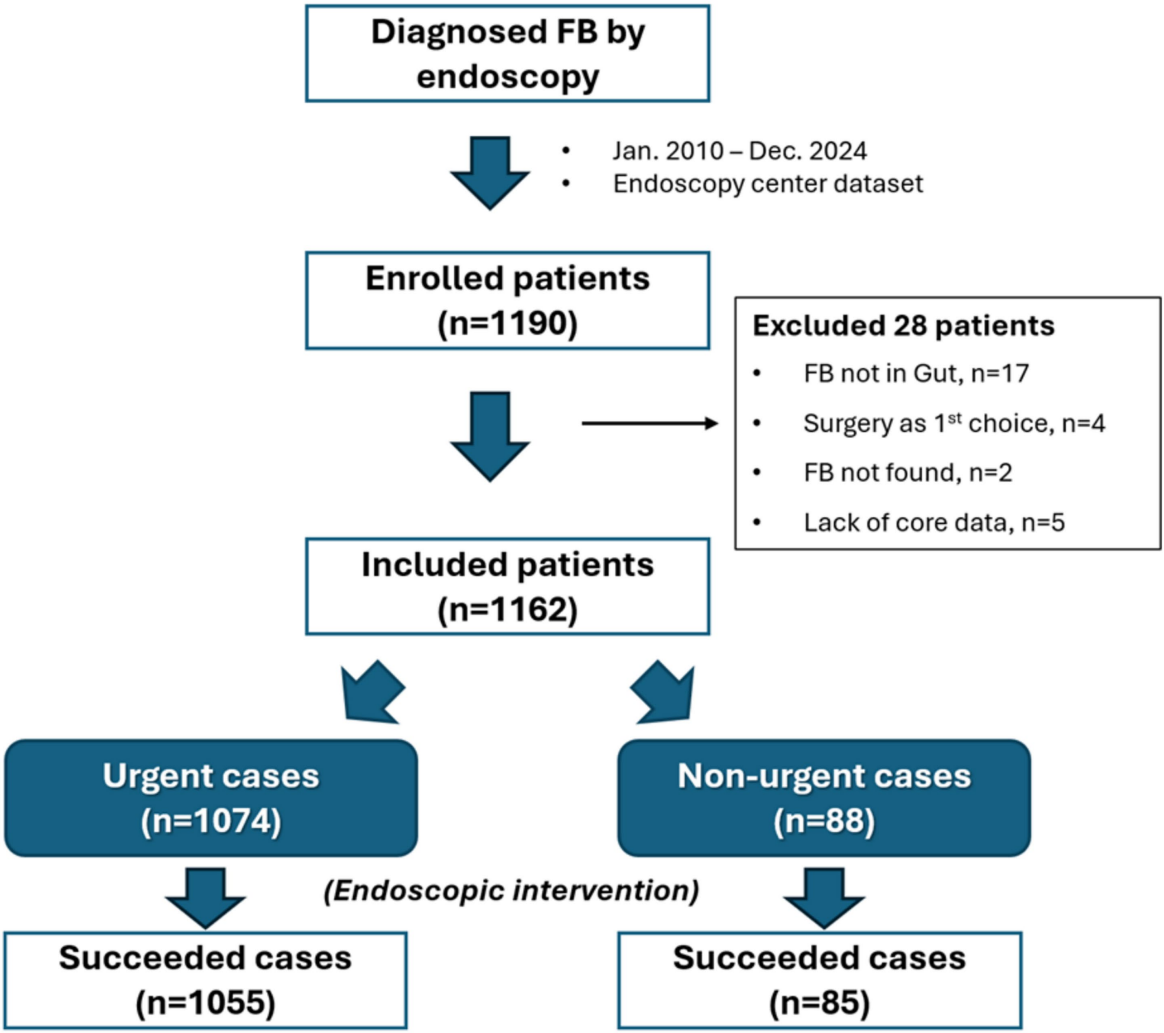
The flow chart and data collection for this study. In sum, 1,162 patients were included for the final analysis, with 28 patients excluded due to our exclusion criteria (white box).

The flow chart of patient selection is summarized in Figure 1. During the 15-year study period, a total of 1,162 cases were enrolled for analysis. Within this cohort, 596 (51.3%) patients were male and 566 (48.7%) were female. Pediatric patients (aged <14 years) accounted for 2.5% (29 cases), adults (aged 15–59 years) accounted for 41.9% (487 cases), and elderly patients (aged ≥60 years) constituted the majority at 55.6% (646 cases). Demographic and clinical characteristics of the cohort are presented in Table 1. The age at FB diagnosis ranged from 1 to 101 years, with a median age of 56 years. The majority of FB stuck in the gut happened accidentally or unconsciously (1,042 cases, 89.7%), whereas intentional ingestion or insertion was documented in 3.3% of cases. Iatrogenic FB impaction was identified in 79 cases (6.8%). The average number of cases occurring on weekends (2 days) was markedly lower than that on weekdays (138 vs. 177, *p* < 0.01). Anatomically, 1,131 cases (97.3%) involved FB impaction within the upper GI tract, while 31 cases (2.7%) occurred in the lower GI tract. Among the 31 lower GI cases, 12 had blunt FBs, 17 had sharp-pointed FBs, and only two had food bolus impactions. Regarding anesthetic approach for these lower GI interventions, sedative colonoscopy was performed in 17 patients, with the remaining 14 patients underwent the procedure under topical anesthesia.

**Table 1:** Demographic and clinical characteristics of patients with foreign body impaction in the alimentary tract.

Variable	No.	Percentage (%)
Total number	1,162	100
Gender (male/female)	596/566	51.3/48.7
Age (years)	56 (median)	1–101 (range)
Primary disease		
Benign/Malignant	1104/58	95.0/5.0
Associated GI stricture	74	6.37
Urgent case	1,074	92.4
Duration of FB impaction	850	73.1
<24 h	715	61.5
24–72 h	51	4.4
72–120 h	14	1.2
>120 h	70	6.0
Location of FB stuck		
Esophagus	1,007	86.7
Stomach	84	7.2
Duodenum	40	3.5
Jejunum or Ileum	4	0.3
Colon	21	1.8
Rectum	5	0.4
Anus	1	0.1
Type of FB		
Blunt	64	5.5
Sharp	1,008	86.7
Food bolus	90	7.8
Size of FB (maximum diameter)		
<1 cm	21	1.8
1–3 cm	894	76.9
>3 cm	247	21.3
Etiology		
Unconscious	1,043	89.8
Intentional	40	3.4
Iatrogenic	79	6.8

GI, gastrointestinal; FB, foreign body.

### Endoscopic outcomes and follow-up results

1,047 cases (90.1%) were managed endoscopically during daytime hours, while 115 cases (9.9%) required emergency intervention at night. Among the 789 cases with documented data regarding the duration from initial FB impaction to the first endoscopic attempt, the median time elapsed was 14 (range: 1.5–13,680) hours.

The frequently used accessory devices for FB extraction were FB forceps and baskets, which occupied 93.1 and 4.9%, respectively. The usage frequencies of other devices, alongside complications secondary to both the FB impaction itself and the endoscopic intervention, are summarized in Table 2. Overall, localized transmural inflammation (35.6%) and mucosal ulceration (41.3%) were the most prevalent pathology findings resulting from FB impaction *in situ*. Following endoscopic intervention, hemorrhage and perforation were each recorded in three cases (0.3%), with no minor mucosal lacerations requiring secondary intervention observed.

**Table 2:** Endoscopic treatment and short-term outcomes of the patients.

Technical index	No.	Percentage (%)
Total number	1,162	100
Anesthesia method		
Topical anesthesia	1,075	92.5
General anesthesia	87	7.5
Time of FB removal		
Day (8:00 ~ 18:00)	1,047	90.1
Night (18:00 ~ 8:00)	115	9.9
Removal method		
FB forceps	1,082	93.1
Biopsy forceps	6	0.5
Snare	41	3.5
Net	1	0.1
Basket	57	4.9
Push into stomach or bowel	5	0.4
Refusal of FB extraction	1	0.1
Complications by FB impaction		
Mild–moderate inflammation	413	35.6
Ulcer	480	41.3
Perforation	97	8.3
Local abscess	98	8.4
Sepsis	74	6.4
Endoscopic complications		
None	1,156	99.4
Hemorrhage	3	0.3
Perforation	3	0.3
Endoscopic outcomes		
Success	1,140	98.1
Transferred to surgery	9	0.8
Others*	13	1.1

* Including emergent ENT operation or endoscopic extraction under general anesthesia in hospitalization. FB, foreign body; ENT, ear nose and throat.

**Figure 2:**
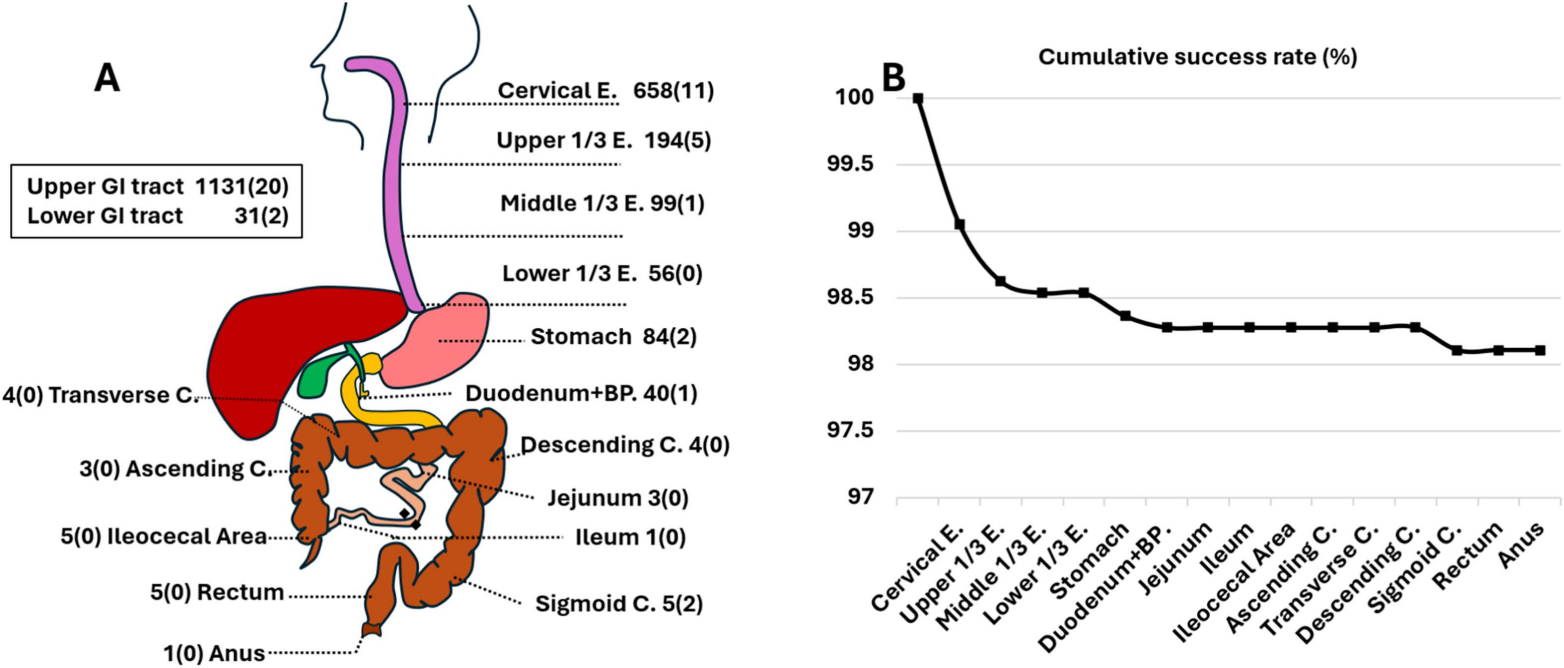
Frequency distribution and cumulative success rates of endoscopic intervention for FB impaction in the GI tract. **(A)** Frequency distribution of cases across specific anatomical segments of the alimentary tract. Values in bracket indicate the absolute number of failed endoscopic interventions within that segment. A dot line in small intestine indicates the boundary between the jejunum and the ileum. **(B)** Cumulative success rate of endoscopic treatment along with the longitudinal GI tract. E., esophagus; BP., biliopancreatic ducts; C., colon; GI, gastrointestinal.

In total, 1,140 cases (98.1%) were successfully managed through endoscopic removal, with 84.7% of the procedures performed within 24 h of suspected FB impaction. Endoscopic treatment was unsuccessful in 22 cases (1.9%), and rescue surgery or ENT procedures under general anesthesia were followed. The anatomical distribution frequency and cumulative success rates of endoscopic FB removal across the alimentary tract are presented in Figure 2, highlighting that the esophagus remains the most challenging site for extraction. Overall, there was no death related to FB impaction or endoscopic treatment in this cohort.

### Risk factors of failure for endoscopic treatment

A multivariate logistic analysis following univariate analysis suggested that an impaction duration exceeding 24 h (*p* = 0.035), sharpness of FB (*p* = 0.014), and multiple endoscopic attempts (*p* < 0.001) are independent risk factors predicting the failure of endoscopic intervention (Table 3).

**Table 3:** Univariate and multivariate regression analyses of risk factors for endoscopic failure of FB removal.

Univariate analysis			Multivariate analysis		
Parameter	No.	*P* value	OR	95%CIs	*P* value
FB duration, >24 h	135	0.001	1.792	1.289 ~ 23.64	0.035
Etiology, intention	119	0.040	1.444	0.467 ~ 4.461	0.523
FB location, esophagus	1,007	0.093	1.123	0.883 ~ 1.428	0.345
FB type, sharpness	1,008	0.031	7.359	1.503 ~ 36.03	0.014
FB size, >3 cm	247	0.186	1.033	0.344 ~ 3.099	0.954
Attempt times, ≥2	76	<0.001	39.95	12.66 ~ 142.8	<0.001

FB, foreign body; OR, odds ratio; CIs, confidence intervals.

## Discussion

In this study, we summarized features of FB impaction and outcomes of endoscopic treatment. We report on the largest single-center cohort in the literature to date, spanning a 15-year period that captured significant technological advancements in therapeutic endoscopy. Crucially, the study is one of few studies to report lower GI outcomes alongside upper alimentary tract data.

An intriguing finding in our cohort was the lower frequency of FB impactions presenting on weekends compared to weekdays, which contrasts with several previous reports (8, 9). It might be explained by the “weekend effect” for inferior emergency management realized by more individuals or by a lower proportion of elderly patients engaging in activities prone to accidental ingestion during weekends, as hypothesized in alternative cohort study (10, 11).

Over the 15-year study period, 31 cases with FB impaction in the lower GI tract (encompassing the small bowel, colon, rectum and anus) were safely managed with endoscopy. Up to the present, it remains great challenging for FB removal from the lower GI tract when considering endoscopic intervention as first choice. Previous consensus guidelines routinely recommended direct referral to colorectal surgery, given that emergency room or endoscopic extractions attempted without deep sedation or general anesthesia frequently failed (12, 13). Conversely, our findings demonstrate that endoscopic retrieval failed in only two cases (6.4%) within this subpopulation (Figure 2). Besides, only four patients with FB impactions in the small bowel underwent primary endoscopic retrieval due to a reachable distance by gastroscope or colonoscope. For others in the middle part of intestine, primary surgical intervention remains the standard of care at our institution, with device-assisted enteroscopy rarely used for FB removal.

The reported success rate of endoscopic FB removal from the upper GI tract was over 95%, with associated complication rate remaining below 5% (1, 14–16). Our institutional data align with these benchmarks, yielding a 98.1% success rate and a low complication rate of 0.6%. Driven by the rapid evolution of endoscopic instrumentation and advanced endoluminal techniques, certain FB embedded partially or entirely into the GI tract can be safely removed with therapeutic endoscopy such as endoscopic submucosal dissection (ESD), endoscopic full-thickness resection (EFTR), and submucosal tunneling endoscopic resection (STER) (17–20). In a way, the endoscopist’s clinical experience and technical proficiency are critical determinants of patient outcomes.

In clinical practice, most patients with GI FB impaction visited emergency department first, thus the timing from diagnosis to endoscopic intervention had to be delayed. Furthermore, the requirement and timing of promptly endoscopic treatment were influenced by many factors, such as patient’s age, comorbidities, FB size and type, anatomical location, complications due to FB impaction, total duration of impaction, and available endoscopic expertise (21–23). By clinical consensus, patients with severe complications due to FB impaction were not fit for endoscopic intervention and should be immediately transferred to intensive care unit or emergent surgical exploration (2). For clinically stable patients with normal vital signs, a rigorous pre-procedural assessment of the risks for aspiration, hemorrhage, bowel obstruction, or perforation is mandatory to determine optimal endoscopic timing. Conversely, asymptomatic, stable patients may be managed conservatively without urgent endoscopy if the ingested FB has a high probability of spontaneous passage or can be safely scheduled for elective removal.

As previously confirmed, duration of FB impaction has been identified as a risk factor for complications, and delayed endoscopic interventions beyond 24 h significantly exacerbate complication rates (14, 24–26). Pathologically, prolonged impaction causes progressive mucosal edema, localized ulceration, and transmural inflammation, which increases tissue fragility and hinders safe endoluminal manipulation (23). Our results also find that duration exceeding 24 h is an independent risk factor of endoscopic failure. Beyond that, additional risk factors including sharpness of FB and multiple endoscopic attempts are detected, which also contributed to increased complication rates (25). Mechanistically, sharp objects could easily embed into the mucosa compared to blunt items and can penetrate into deeper layers of alimentary tract driven by physiological peristalsis. It makes grasping technically difficult and increases perforation risk during withdrawal. Furthermore, multiple retrieval attempts typically reflect highly challenging clinical scenarios, such as poorly grasped due to large size, deep penetration due to sharp edges, and difficult location for endoscope arrival (27). Repetitive manipulation prolongs procedure times, thereby multiplying tissue trauma and the risk of iatrogenic perforation (14). Interestingly, while an absolute FB size exceeding 3 cm has been cited as a predictor for surgical conversion in prior literature (28), it did not reach statistical significance in our study. In clinical practice, maximal diameter of FB was estimated mostly by operative endoscopist. Moreover, the spatial orientation of the impacted object in the GI lumen is functionally more critical than its absolute size, particularly in the esophagus where the cricopharyngeal and lower esophageal sphincter create fixed anatomical constraints.

Several limitations inherent to the retrospective design of this study must be acknowledged. First, the study period spanned 15 years, during which temporal advancements in endoscopy likely influenced clinical outcomes; notably, 16 of the 22 failed cases (72.7%) occurred prior to 2017. Besides, cases were first handled by ENT surgeons with rigid laryngoscope or oesophagoscope were not included. Second, our analysis is mainly based on a retrospective review of available cases from endoscopic datasets and medical records, so the duration prior to endoscopic treatment and degree of complications were not defined objectively. The cohort included a small number of pediatric patients, limiting the generalizability of these findings to broader pediatric populations. Additionally, granular data regarding precise procedural durations and individual endoscopist experience levels were unavailable, introducing potential confounding bias. Third, due to the small number of cases with major complications, we failed to analyze the predicting factors or risk ratios for severe complications. Finally, most cases (92.5%) received topical anesthesia, with no correlation found between the anesthesia method and endoscopic complications. Although previous study by Geng et al. suggest that general anesthesia yields lower complication rates than topical anesthesia (1), our study was limited in its statistical power to evaluate this. Prospective, well-powered multicenter trials are required to fully elucidate the impact of various anesthetic modalities on optimizing GI foreign body retrieval.

In conclusion, patients with FB stuck in the alimentary tract can be safely managed with endoscopic removal, with a success rate of 98.1% observed in this large cohort. Endoscopic management appears feasible even for complex cases involving the lower GI when performed at experienced centers, warranting further prospective study. Esophagus, particularly its proximal segment, remains a challenging zone for endoscopic extraction. More importantly, risk factors including delayed endoscopic removal exceeding 24 h, sharp-pointed objects and the need for multiple endoscopic attempts should be early recognized to prevent failure of endoscopic intervention.

## Data Availability

The raw data supporting the conclusions of this article will be made available by the authors, without undue reservation.

## References

[1] Geng C Li X Luo R Cai L Lei X Wang C. Endoscopic management of foreign bodies in the upper gastrointestinal tract: a retrospective study of 1294 cases. Scand J Gastroenterol. (2017) 52:1286–91. doi: 10.1080/00365521.2017.1350284, 28691540

[2] Birk M Bauerfeind P Deprez P Häfner M Hartmann D Hassan C . Removal of foreign bodies in the upper gastrointestinal tract in adults: european society of gastrointestinal endoscopy (ESGE) clinical guideline. Endoscopy. (2016) 48:489–96. doi: 10.1055/s-0042-100456, 26862844

[3] Loria A Marianetti I Cook CA Melucci AD Ghaffar A Juviler P . Epidemiology and healthcare utilization for rectal foreign bodies in United States adults, 2012–2021. Am J Emerg Med. (2023) 69:76–82. doi: 10.1016/j.ajem.2023.03.041, 37060632

[4] Mckechnie JC. Gastroscopic removal of a phytobezoar. Gastroenterology. (1972) 62:1047–51. doi: 10.1016/S0016-5085(72)80123-9, 5029071

[5] Webb WA. Management of foreign bodies of the upper gastrointestinal tract. Gastroenterology. (1988) 94:204–16. doi: 10.1016/0016-5085(88)90632-4, 3275566

[6] Conway WC Sugawa C Ono H Lucas CE. Upper GI foreign body: an adult urban emergency hospital experience. Surg Endosc. (2007) 21:455–60. doi: 10.1007/s00464-006-9004-z, 17131048

[7] Zhang S Cui Y Gong X Gu F Chen M Zhong B. Endoscopic management of foreign bodies in the upper gastrointestinal tract in South China: a retrospective study of 561 cases. Dig Dis Sci. (2010) 55:1305–12. doi: 10.1007/s10620-009-0900-7, 19655249

[8] Wu L Lei G Liu Y Wei Z Yin Y Li Y . Retrospective analysis of esophageal foreign body ingestion: differences among weekday, weekends, and holidays. Risk Manag Healthc Policy. (2021) 14:2499–506. doi: 10.2147/RMHP.S314069, 34163269 PMC8214566

[9] Shah SR Little DC. "Ingested and aspirated foreign bodies". In: Holcomb and Ashcraft’s Pediatric Surgery. Eds. St. Peter S. D. Snyder C. L.. Philadelphia, PA: Elsevier (2026). p. 144–54.

[10] Zapf MAC Kothari AN Markossian T Gupta GN Blackwell RH Wai PY . The “weekend effect” in urgent general operative procedures. Surgery. (2015) 158:508–14. doi: 10.1016/j.surg.2015.02.024, 26013983 PMC5226376

[11] Zhong Q Jiang R Zheng X Xu G Fan X Xu Y . Esophageal foreign body ingestion in adults on weekdays and holidays: a retrospective study of 1058 patients. Medicine (Baltimore). (2017) 96:e8409. doi: 10.1097/MD.0000000000008409, 29069038 PMC5671871

[12] Clarke DL Buccimazza I Anderson FA Thomson SR. Colorectal foreign bodies. Color Dis. (2005) 7:98–103. doi: 10.1111/j.1463-1318.2004.00699.x, 15606596

[13] Coskun A Erkan N Yakan S Yıldirim M Cengiz F. Management of rectal foreign bodies. World J Emerg Surg. (2013) 8:11. doi: 10.1186/1749-7922-8-11, 23497492 PMC3601006

[14] Hong KH. Risk factors for complications associated with upper gastrointestinal foreign bodies. World J Gastroenterol. (2015) 21:8125–31. doi: 10.3748/wjg.v21.i26.8125, 26185385 PMC4499356

[15] Limpias Kamiya KJ Hosoe N Takabayashi K Kamiya KJLL Hayashi Y Sun X . Endoscopic removal of foreign bodies: a retrospective study in Japan. World J Gastrointest Endosc. (2020) 12:33–41. doi: 10.4253/wjge.v12.i1.33, 31942232 PMC6939123

[16] Yoo DR Im CB Jun BG Seo HI Park JK Lee SJ . Clinical outcomes of endoscopic removal of foreign bodies from the upper gastrointestinal tract. BMC Gastroenterol. (2021) 21:385. doi: 10.1186/s12876-021-01959-3, 34666708 PMC8524826

[17] Carvalho AC Pires F Araújo R. Removal of an embedded foreign body in the stomach by a technique of endoscopic submucosal dissection. Dig Endosc. (2022) 34:34. doi: 10.1111/den.14132, 34555869

[18] Ma L-Y Liu Z-Q Chen W-F Zhong YS Li QL Zhou PH. Endoscopic removal of a perforating and embedded foreign body in the duodenum. Am J Gastroenterol. (2022) 117:1560–08. doi: 10.14309/ajg.0000000000001823, 35509123

[19] Su W Jiang Q Yang X Wang Y Hu J Gao P . The safety and efficacy of endoscopic submucosal fenestration in the treatment of completely embedded upper gastrointestinal foreign body. Surg Endosc. (2024) 38:3819–27. doi: 10.1007/s00464-024-10899-4, 38811429

[20] Qin L Shi H Zhang X Chen P Liu X Wang J . Endoscopic removal of esophageal foreign body embedded in muscularis propria. Endoscopy. (2024) 56:E89–90. doi: 10.1055/a-2239-3296, 38290708 PMC10827518

[21] Ciriza C García L Suárez P Jiménez C Romero MJ Urquiza O . What predictive parameters best indicate the need for emergent gastrointestinal endoscopy after foreign body ingestion? J Clin Gastroenterol. (2000) 31:23–8. doi: 10.1097/00004836-200007000-00006, 10914771

[22] Mosca S Manes G Martino R Amitrano L Bottino V Bove A . Endoscopic management of foreign bodies in the upper gastrointestinal tract: report on a series of 414 adult patients. Endoscopy. (2001) 33:692–6. doi: 10.1055/s-2001-16212, 11490386

[23] Ikenberry SO Jue TL Anderson MA Appalaneni V Banerjee S Ben-Menachem T . Management of ingested foreign bodies and food impactions. Gastrointest Endosc. (2011) 73:1085–91. doi: 10.1016/j.gie.2010.11.010, 21628009

[24] Lai ATY Chow TL Lee DTY Kwok SPY. Risk factors predicting the development of complications after foreign body ingestion. Br J Surg. (2003) 90:1531–5. doi: 10.1002/bjs.4356, 14648732

[25] Sung SH Jeon SW Son HS Kim SK Jung MK Cho CM . Factors predictive of risk for complications in patients with oesophageal foreign bodies. Dig Liver Dis. (2011) 43:632–5. doi: 10.1016/j.dld.2011.02.018, 21466978

[26] Hung C-W Hung S-C Lee CJ Lee WH Wu KH. Risk factors for complications after a foreign body is retained in the esophagus. J Emerg Med. (2012) 43:423–7. doi: 10.1016/j.jemermed.2011.01.030, 21669509

[27] Alotaibi SM Alobaida NW Aljomah DS AlShahrani M Binnasser A. Removal of large foreign body from airway via combined endoscopic and open approach: a case report and literature review. Otolaryngol Case Rep. (2022) 23:100417. doi: 10.1016/j.xocr.2022.100417, 38826717

[28] Lee H-J Kim H-S Jeon J Park SH Lim SU Jun CH . Endoscopic foreign body removal in the upper gastrointestinal tract: risk factors predicting conversion to surgery. Surg Endosc. (2016) 30:106–13. doi: 10.1007/s00464-015-4167-0, 25805240

