# Supplementary material for: Endoscopic treatment outcomes for foreign body extraction from alimentary tract: a 15-year experience of 1,162 cases in Southern China
Source: Front Med (Lausanne). 2026 Jul 1;13:1877577. doi: 10.3389/fmed.2026.1877577 (PMC13368547; doi:10.3389/fmed.2026.1877577)
Supplement: Supplementary file 1 [file Supplementary_file_1.docx]

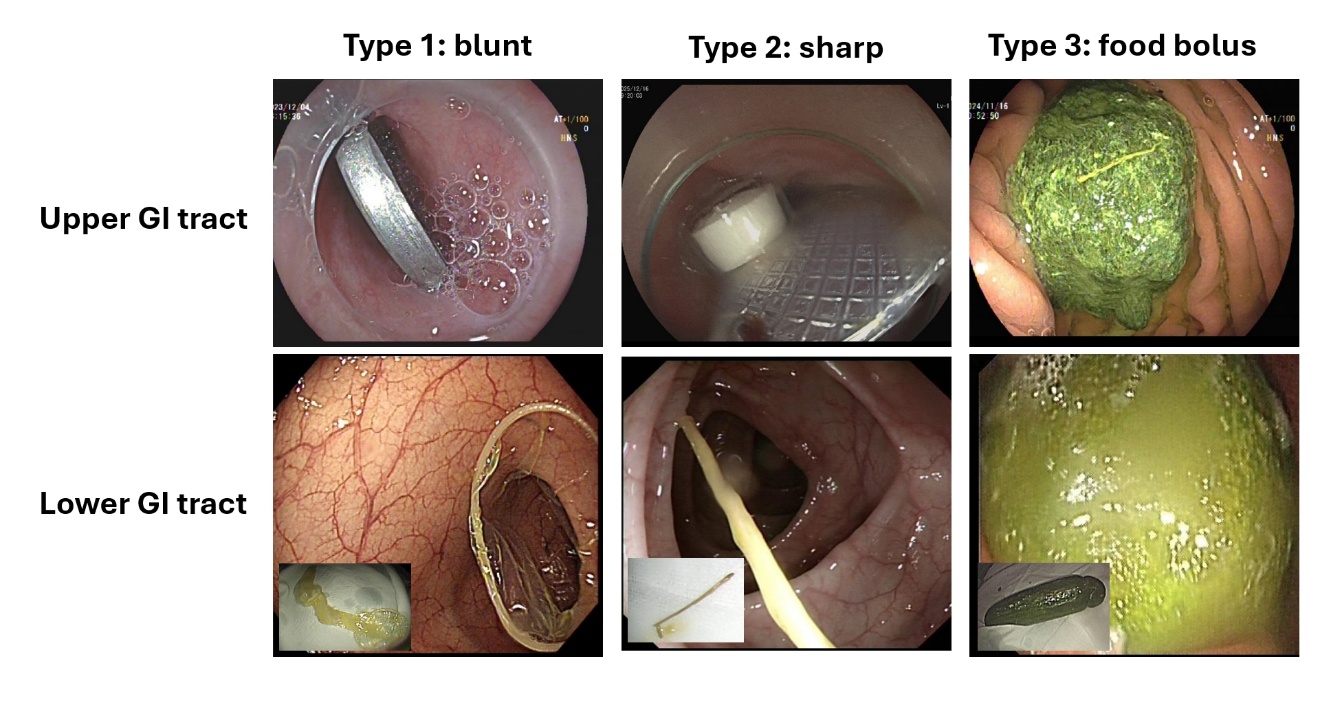


**Appendix Figure 1.** Typical images indicating three types of foreign bodies stuck in both upper and lower gastrointestinal tracts.

All six images were selected from the enrolled patients who confirmed diagnosis of foreign body impaction in the alimentary tract during endoscopic procedure. Abbreviation: GI, gastrointestinal.
